# Financial toxicity and employment status in cancer survivors. A systematic literature review

**DOI:** 10.1007/s00520-020-05719-z

**Published:** 2020-08-31

**Authors:** Floortje Mols, Bianca Tomalin, Alison Pearce, Billingsley Kaambwa, Bogda Koczwara

**Affiliations:** 1 CoRPS—Center of Research on Psychological and Somatic Disorders, Department of Medical and Clinical Psychology, Tilburg University, Tilburg, The Netherlands; 2 Department of Research, Netherlands Comprehensive Cancer Organisation (IKNL), Utrecht, The Netherlands; 3 Flinders Centre for Innovation in Cancer (FCIC), Flinders Medical Centre and Flinders University, Adelaide, SA Australia; 4 Centre for Health Economics Research and Evaluations, University of Technology Sydney, Sydney, NSW Australia; 5 Health Economics, Flinders University, Adelaide, SA Australia; 6 Sydney School of Public Health, University of Sydney, Sydney, NSW Australia

**Keywords:** Financial toxicity, Employment, Cancer survivors, Costs

## Abstract

**Background:**

Financial toxicity has traditionally been attributed to the rising costs of cancer care. As ability to work impacts one’s financial situation, limited employment and reduced income may also contribute to financial toxicity. We examined evidence of the association between financial toxicity and employment status in cancer survivors.

**Methods:**

A systematic literature review was performed via PubMed, Web of Science, CINAHL, and PsycINFO with search terms including “Cancer,” “Financial toxicity,” and “Employment” on September 25, 2019.

**Results:**

Thirty-one papers met eligibility criteria. Thirteen studies were rated as having high quality, 16 as adequate, and two as low. Being actively treated for cancer had serious negative consequences on employment and medical expenditures. Unemployment, changed or reduced employment, lost days at work, poor work ability, and changes to employment were associated with a higher risk of financial toxicity. Patients who were younger, non-white, unmarried, of low education, living with dependents, residing in non-metropolitan service areas, with lower income, and of low socioeconomic status were more at risk of financial toxicity. Other variables associated with financial toxicity included having a mortgage/personal loan, higher out of pocket costs and household bills, limited health insurance, more severely ill, on active treatment, and lower functioning or quality of life.

**Conclusion:**

Cancer negatively affects employment, and these changes are significant contributors to financial toxicity. Researchers, healthcare professionals, and patients themselves should all cooperate to tackle these complex issues.

## Introduction

Financial toxicity refers to the financial burden or financial hardship experienced by cancer survivors because of cancer and its treatment [1–3]. The problem of financial toxicity is increasing since the costs of care are increasing with newer treatments, the prevalence of cancer is growing rapidly, and many survivors live with cancer as a chronic disease. Depending on the country and thus the healthcare system, financial toxicity prevalence varies widely, but studies have shown consistently that its presence is associated with lower quality of life, poorer adherence to or delay of care, and early mortality [4–7].

High costs of cancer care are a recognized cause of financial toxicity through medical costs (such as cost of new treatments), non-medical costs (e.g., travel costs to hospitals), or indirect costs (e.g., lost wages as a result of time off work for cancer treatment) [5]. Even if healthcare is available to everyone via universal health insurance coverage, patients have out-of-pocket expenses (OOP) in relation to their disease and its treatment. Since many cancer survivors are known to experience long-term side effects and symptoms of cancer and its treatment, these costs can continue even years after diagnosis [8].

To date, a number of systematic reviews have examined financial toxicity in cancer survivors [2, 9, 10]. A recent review summarized 45 studies and concluded that 47–49% of cancer survivors reported some degree of financial distress [9]. Another recent review examined 25 studies from nine countries with the majority from the USA and showed that up to 73% of patients reported financial toxicity [2]. Predictors of financial toxicity included younger age, female gender, a more recent diagnosis, and use of adjuvant therapies [2]. A review that focused on the relationship between financial toxicity and symptom burden concluded that a clear association exists between financial toxicity and psychological symptoms like depression [10].

While the focus on financial toxicity has historically been on the costs of cancer care, especially in light of the significant rise in the cost of cancer medicine [11], limitations in or inability to work is also likely to contribute to financial toxicity [2, 9]. Both income and changes in work participation have been associated with financial toxicity [2]. Similarly, reduced income and missed days of work due to illness are associated with financial hardship [9]. Data on employment after cancer show that as many as 40% of employed cancer survivors do not return to work after cancer diagnosis [12], and inability to work is associated with greater financial hardship and reduced quality of life [6]. Those more likely to return to work after diagnosis are individuals with a higher educational level, male gender, and younger age at diagnosis; those that underwent less invasive surgery, experienced fewer physical symptoms, and had a lower length of sick leave; and those with provision of workplace accommodations such as flexible hours or rehabilitation services, lower length of sick leave and continuity of care [13]. This significant overlap between predictors of financial toxicity and predictors of unemployment after cancer raises the question of how employment status and financial toxicity after cancer are related, taking into account a possibility of confounding.

To address this question, the primary aim of this systematic literature review was to examine the relationship between financial toxicity and employment in cancer survivors and any variables that may affect this relationship.

## Methods

### Search strategy

We followed the Preferred Reporting Items for Systematic Reviews and Meta-Analyses (PRISMA) guidelines [14]. A computerized search of the literature through the search engines PubMed, Web of Science, CINAHL, and PsycINFO was performed on September 25, 2019. The search terms captured concepts of “financial toxicity,” “employment,” and “cancer.” Boolean operators and keywords were used with Medical Subject Headings (MeSH) where possible. Separate searches were conducted for each database. All search results were imported in EndNote, which was used to remove duplicates. Reference lists of all identified publications were checked to retrieve other relevant publications not identified by means of the computerized search.

### Inclusion and exclusion criteria

Studies that met the following criteria were included: (1) if the objective was to describe financial toxicity and employment in adult cancer survivors, (2) if the publication described a quantitative study, (3) if the publication was an original article (e.g., no poster abstracts, editorials, reviews, and letters to the editor), (4) if they were published or in press in peer-review journals, and (5) if they were written in English. Studies were excluded for the following reasons: (1) if they included participants under the age of 18; (2) if they focused solely on spouses, caregivers, family of cancer survivors, or health professionals; or (3) if they included patients with other diseases besides cancer as well.

### Screening

**Fig. 1:**
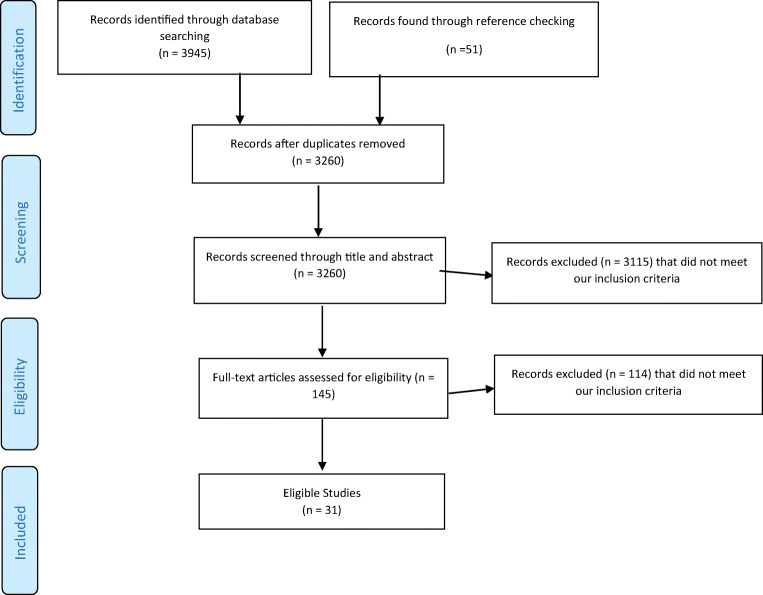
PRISMA flow diagram

Articles were reviewed by title and abstract according to the pre-specified inclusion and exclusion criteria. Then full-text papers were reviewed to confirm eligibility. Results of the search were discussed, and any discrepancies were clarified until consensus was reached. A flowchart of this selection procedure is shown in Fig. 1.

### Quality assessment

The methodological quality of all included articles was assessed according to predefined criteria using a 13-item standardized checklist. The checklist was a slightly adapted version of an established criteria list for systematic reviews [15, 16]. The criteria are presented in Table 1.

**Table 1:** List of criteria for assessing the methodological quality of studies on the association between financial toxicity and employment status

	Criteria	Number of articles meeting criteria (*n* = 31)
	Positive if with respect to	
	*Main outcomes*	
1	A validated financial toxicity questionnaire is used	11
2	Financial toxicity was assessed objectively	2
	*Study population*	
3	A description is included of at least two socio-demographic variables	31
4	A description is included of at least two clinical variables	30
5	Inclusion and/or exclusion criteria are described	28
6	Participation rates for patient groups are described and are > 70%	10
7	Information is given about the degree of selection of sample (e.g., whether there is a selective response)	9
	*Study design*	
8	The study size is consisting of at least 50 participants (arbitrarily chosen)	31
9.	The collection of data is prospectively gathered	8
10.	The process of data collection is described (e.g. interview or self-report)	30
	*Results*	
11.	The results are compared between two groups or more (e.g., comparison with healthy population and differences in financial toxicity between those with or without work), and/or results are compared between at least two time points (e.g., pre- versus post-treatment)	30
12.	Statistical proof for the main findings is reported	30
13.	Relationship between financial toxicity and employment status is described	23

Each item of an article that matched our criteria received one point. Zero points were assigned if an item did not meet our criteria, was described insufficiently, or not at all. The highest possible score was 13. Studies scoring ≥ 75% of the maximum attainable score (≥ 10 points) were, arbitrarily, considered to be of “high quality.” Studies scoring between 50 and 75% (7–9 points) were rated as “adequate quality.” Studies scoring lower than 50% (i.e., < 7 points) of the maximum attainable score were considered to be of “low quality.”

## Results

### Study characteristics

The search identified 3945 unique citations (PubMed (*n* = 2891), Web of Science (*n* = 775), CINAHL (*n* = 180), and PsycINFO (*n* = 99)) with 31 studies meeting our inclusion criteria (Table 2) [8, 17–43]. All were published between 1990 and 2019 and originated from the USA (*N* = 16), Australia (*N* = 6), the Netherlands (*N* = 2), Canada (n = 1), Japan (*N* = 1), Singapore (*N* = 1), Iran (*N* = 1), the UK (*N* = 1), Germany (*N* = 1), or Ireland (*N* = 1). A total of 16 studies reported on data from various tumors [8, 18, 20, 21, 26, 29–33, 36–38, 40, 41, 43], 5 studies focused on breast cancer [19, 25, 28, 35, 42, 44] and 2 included multiple myeloma patients [22, 45]. The other studies focused on bladder [39], prostate [23], colorectal [24], lung [27], head and neck cancer [34], and bone marrow transplant patients [17]. Time since diagnosis ranged from a mean of 8.4 months before diagnosis [27] until a mean of 13 years after diagnosis [19] often had a broad range, and sometimes was not reported at all. Sample sizes ranged from 129 [19] to 16,771 [30] participants. Eight studies had a longitudinal design [19, 24, 25, 27, 32, 35, 42, 44].

**Table 2:** Characteristics and quality assessment of eligible studies

Authors/year/country	Study design	Sample size and population	Time since diagnosis	Objectives	Financial toxicity outcome measures used	Main findings	Quality assessment
Albelda 2019 USA [17]	Cross-sectional	*N* = 171 Bone marrow transplant	6 months	Effects of access to paid leave on health, QOL, perceived stress, and reduction in financial burden of employed patients who underwent bone marrow transplantation	A 43-item survey developed after a structured literature review, focus group, consultations with researchers, and 7 in-depth interviews with patients	Paid leave affected health outcomes mostly through alleviating financial burden	8
Arndt 2019 Germany [18]	Cross-sectional, Population-based	*N* = 1.158 Mixed	Mean = 8.3 year	How many survivors return to work and when? How many make work changes (content, hours, quit) and is this related to financial toxicity? Does proportion of return to work vary according to certain characteristics?	EORTC QLQ-C30 financial difficulties item	Most survivors return to work. This was related to age at diagnosis, tumor stage, education and occupational class. Financial problems may arise due to reduced work hours	10
Banegas 2016 USA [38]	Cross-sectional	*N* = 4.719 Mixed	<1 yr; *N* = 940 1–4 yr, *N* = 1.845 5-9 yrs., *N* = 822 ≥10 yrs., *N* = 1026	To assess the proportion of working-age cancer survivors who reported that they or their families had gone into debt and the proportion who reported having filed for bankruptcy because of cancer, its treatment, or the lasting effects of that treatment	Amount borrowed and size of debt incurred, worry about paying bills, making financial sacrifices, types of OOP expenses	Younger, unemployed, and uninsured or publicly insured working-age survivors were at greater risk for debt (OR = 1.28, *p* < 0.05) and bankruptcy (OR = 1.74, *p* < 0.05)	9
Casilla-Lennon, 2018 USA [39]	Cross-sectional	*N* = 138 Bladder cancer	Median (IQR) = 0.4 (0.1, 1,2)	To define the prevalence of financial toxicity among bladder cancer patients and identify delays in care and its effect of HRQOL	Selecting “agree” or “strongly agree” on the following statement; “You have to pay more for medical care than you can afford”	Financial toxicity is a major concern, especially among younger bladder cancer patients. Whether the patient works for pay was not associated with financial toxicity. Patients reporting financial toxicity were more likely to delay care due to factors like inability to take time of work (*p* = 0.04)	10
Dean 2019 USA [19]	Retrospective Prospective	*N* = 129 Breast cancer	Mean = 12 year	Compare OOP costs for breast cancer survivors with and without lymphedema	Goosens’ cost diary, Breast Cancer Finances Survey	A significant greater percentage of women with lymphedema were in a lower income category. Breast cancer sometimes influenced work opportunities and long-term earning potential, and breast cancer-related lymphedema may further decrease productivity losses at work	10
Finkelstein 2009 USA [20]	Cross-sectional	*N* = 1940 Mixed	Not reported	To present nationally representative estimates of the effect of cancer care on OOP medical expenditures and lost productivity for the working-aged population	Medical Expenditure Panel Survey; total annual medical spending, including insurance and annual OOP spending	Cancer treatment increases the mean annual OOP medical expenditures by $1170. Those undergoing active cancer care were less likely to be employed full-time. Those employed undergoing cancer care missed 22.3 more workdays a year compared with those without cancer	5
Authors/year/country	Study design	Sample size and population	Time since diagnosis	Objectives	Financial toxicity outcome measures used	Main findings	Quality assessment
Ghasempour 2015 Iran [21]	Cross-sectional	*N* = 165 Mixed	Mean = 36.5 months	To determine the rate of return to work and its relation to financial distress among Iranian cancer survivors	Financial distress/financial well-being scale	Financial distress was significantly lower among survivors who had returned completely to work in comparison with patients who had quit working for cancer-related reasons or returned to work as part-time workers	10
Goodwin 2013 USA [22]	Cross-sectional	*N* = 762 Multiple myeloma	Mean = 5 years since treatment	To identify the personal financial effects of cancer among a cohort of multiple myeloma patients and its treatment in 5 areas including disability, employment, retirement, insurance (Health/medical and life), and OOP expenses	Self-designed questionnaire on employment, disability, health/medical and life insurance, retirement, and OOP expenses related to treatment	High OOP costs were reported among those with a reduced income from less worked hours; they also mention insurance coverage among unemployed individuals contributing to financial toxicity	9
Gordon 2017 Australia [23]	Cross-sectional	*N* = 289 Prostate cancer	*N* = 132 diagnosed < 3 years ago; *N* = 189 diagnosed < 5 years ago	To understand the extent, nature and variability of the current economic burden of prostate cancer among Australian men	Self-designed questionnaire covering, among others, the following domains; employment, household finances, OOP expenses of prostate cancer, and private health insurance status.	20% of men reported that the cost of treatment caused them “a great deal” of distress. Respondents in paid employment at diagnosis stated that they had retired 4–5 years earlier than planned	7
Gordon 2017 Australia [24]	Prospective POPULATION-based	*N* = 187 Colorectal cancer	6 and 12 months after diagnosis	To gain a better understanding of the work situation and the financial wellbeing of colorectal cancer survivors during the 12 months following diagnosis	3 financial toxicity measures: financial strain, ability to raise money, and perceived prosperity. “Increased work” and “reduced work” were defined as > 4 h difference per week	Middle-aged working cancer survivors were 66% more likely (*p* < 0.05) to report not being financially comfortable if they had ceased/decreased employment (21% or 33%) compared with those who maintained/increased employment participation (15% or 19%)	10
Gordon 2007 Australia [25]	Longitudinal Population-based	*N* = 287 Breast cancer	6, 9, 12, 15, 18 months	Identify and describe the direct and indirect economic losses to breast cancer survivors in Australia	Self-designed financial toxicity questionnaire on health service expenditure, physical and social support programs, use and cost of domestic services, care-giving support, OOP expenses, (un)paid work reductions and lost income	Economic costs related to breast cancer may continue to affect women 18 months post-diagnosis. Lost income, health services expenditures, and lost unpaid work were the greatest sources of economic burden	10
Authors/year/country	Study design	Sample size and population	Time since diagnosis	Objectives	Financial toxicity outcome measures used	Main findings	Quality assessment
Graeves-Otte 1990 Netherlands [26]	Cross-sectional	*N* = 849 Mixed	7–9 years ago	To what degree do impediments occur in everyday activities; what, if any, problems exist when a patient returns to a previous job or tries a new job; and what insurance problems are there?	Self-designed financial toxicity questionnaire	Compared with before diagnosis, the socioeconomic position had not changed in 62%. 28% of respondents employed at diagnosis were now housekeepers (99% female). Absence from work at survey did not differ from absence in the year before diagnosis. Cancer had a negative impact on promotional aspects, income and getting insurance	8
Guerin 2016 USA [27]	Retrospective follow-up study	*N* = 132 Lung cancer with brain metastasis	A median of 8.4 and 6.6 months in the pre- and post-diagnosis periods	To investigate healthcare utilization, direct and indirect costs of care among lung cancer patients with brain metastasis	A large administrative-claims database (Optum health reporting and insights database), productivity loss data, salary information	Development of brain metastasis in lung cancer patients is associated with a substantial economic burden to payers, patients and employers	10
Guy 2015 USA [8]	Cross-sectional	*N* = 4.960 Mixed	*N* = 348 ≤ 1 years after diagnosis; *N* = 1.993 > 1 year after diagnosis	To present nationally representative estimates of the impact of cancer survivorship on medical expenditures and lost productivity among US adults	MEPS, direct medical costs were estimated using total annual medical expenditures, by source of payment and service type	Cancer survivors aged 18 to 64 years were more likely to report employment disability, an higher number of missed workdays because of health, and higher number of additional days spent in bed because of health than individuals without a history of cancer	6
Honda 2019 Japan [40]	Cross-sectional	*N* = 156 Mixed solid tumors	Time to first chemotherapy = median 12 months (2–138 months)	To describe the burden and characteristics of financial toxicity among Japanese patients with cancer	The COST score, OOP medical costs, total family income, and total family savings	Older age and higher household savings were negatively associated with financial toxicity; non-regular employment, retirement because of cancer, and use of strategies to cope with the cost of cancer care were negatively associated with COST score	10
Huntington 2015 USA [45]	Cross-sectional	*N* = 100 Multiple myeloma	31 months median	To measure financial toxicity and its effects on patients undergoing treatment for multiple myeloma	COST measure.	71% reported at least minor financial burden, 36% reported applying for financial assistance. Use of savings for treatment was common (46%) and 21% borrowed money for medication. Stopping with work since diagnosis lead to more financial toxicity	10
Jagsi 2014 USA [44]	Longitudinal prospective	*N* = 1.502 Breast cancer	Mean of 9 months after diagnosis and 4 years later	To evaluate the financial experiences of a group of racially and ethnically diverse cohort of long-term breast cancer survivors	Several measures for financial impact, Perception of financial decline, OOP costs, employment and insurance	33% reported financial decline since diagnosis. Among others, younger than 65, household income < $50,000 at diagnosis, part-time employment at diagnosis, and reduction in work hours attributed to financial decline	10
Joo Ho 2018 Singapore [28]	Cross-sectional	*N* = 327 Breast cancer	Employed = 4 years median, Unemployed = 5 years median	To explore the determinants of employment and suboptimal workability and evaluate the association between workability and patient-reported physical, psychological, and social outcomes	Workability index, EORTC QLC-C30	Lower employment and reduced workability in breast cancer survivors is common, and reduced workability is associated with higher levels of depression, financial difficulty, physical fatigue, more breast symptoms, and poorer global health status	10
Authors/year/country	Study design	Sample size and population	Time since diagnosis	Objectives	Financial toxicity outcome measures used	Main findings	**Quality assessment**
Khera 2014 USA [29]	Cross-sectional, retrospective	*N* = 268 Allogeneic HCT patients	2.3 years median	To describe the impact of financial burden on health behaviors and evaluate the risk factors for objective financial burden and treatment non-adherence	Self-designed questionnaire on subjective and objective financial burden along with OOP expenses, medication copayments, insurance, and income	Forty-seven percent experienced financial burden. Younger age and poor current mental and physical functioning increased the likelihood of financial burden. Thirty-five percent reported deleterious health behaviors because of financial constraints. Being employed decreased the likelihood of experiencing financial burden and treatment non-adherence due to concern about costs	9
Lauzier 2013 Canada [42]	Longitudinal prospective	*N* = 829 Breast cancer	1, 6 and 12 months after start of treatment	To assess OOP costs and wage losses during the first year after diagnosis	All OOP costs including costs associated with surgeries, adjuvant treatments, complications, follow-up visits, transportation costs, parking costs, and meal costs	Higher OOP costs were associated with higher education, working at diagnosis, living > 50 km from the hospital, and having multiple types of adjuvant treatment. When considered simultaneously with wage losses, OOP costs were not associated with perceived deterioration in the family’s financial situation; rather, wage losses were the driving factor	11
Massa 2019 USA [30]	Cross-sectional	*N* = 16.771 Mixed	Not reported	To assess the financial burdens of patients with head and neck cancer compared with other cancers	MEPS database using household and condition survey files	Medical expenses and relative OOP costs were higher for head and neck cancer patients than for patients with other cancers. Higher OOP expenses were associated with unemployment, public insurance, poverty, and lower health status	9
Nekhlyudov 2016 USA [31]	Cross-sectional	*N* = 615 Mixed	5–6 years, *N* = 240 7–8 years, *N* = 213 9–10 years, *N* = 162	To examine how insurance coverage, financial status, and employment vary for survivors of different cancer types	Medical Expenditures Panel Survey (MEPS)—Experiences with Cancer Survivorship Survey	Fifteen percent experienced financial hardship due to cancer. Twenty-five percent of those employed reported that they or their spouses remained at their jobs due to concerns about losing medical insurance. Sixty-three percent reported making changes in their jobs due to cancer, and 42% reported that cancer interfered with their tasks at work or reduced productivity. Negative employment and financial implications were most common among those with colorectal, lung, and breast cancer, and those diagnosed before age 65	9
Paul 2016 Australia [32]	Cross-sectional	*N* = 255 Mixed	≤ 12 months, *N* = 113 > 12 months, *N* = 139	Quantify effects on income and employment; describe how cost-related factors influence treatment decision-making and need for financial assistance; identify patient factors associated with treatment decision-making, use of financial assistance	Self-designed questionnaire on employment, income, financial assistance, and treatment decision-making	Sixty-seven percent indicated a change in employment and 63% of those reported reduced household income since diagnosis. Unemployment status had higher odds of patients reporting reduced income (23.1%, 95%CI: 14.8–31.3). Travel (15%), loss of income (14%), and cost of treatments (11%) were factors influencing treatment decision-making	10
Authors/year/country	Study design	Sample size and population	Time since diagnosis	Objectives	Financial toxicity outcome measures used	Main findings	**Quality assessment**
Pearce 2019 Australia [33]	Cross-sectional	*N* = 2.931 Mixed	Mean 3.7 year (SD = 3.4), median 3 year	To examine the relationship between employment and financial toxicity by examining the prevalence of, and factors associated with, financial toxicity among cancer survivors	A question from the EORTC QLQ-C30 “Has your physical condition or medical treatment caused you financial difficulties in the past week?”	Forty-nine percent of survivors were employed, and 22% reported financial toxicity. Those not employed were at greater risk of financial toxicity (27% vs 16%). The odds of reporting financial toxicity were greater for those who were male, younger, unmarried, with low education, low socioeconomic status, or without paid employment	9
Rogers 2012 UK [34]	Cross-sectional	*N* = 447 Head and neck cancer	Months since treatment; < 12; *N* = 169 12–23; *N* = 155 24–59; *N* = 244 60+, *N* = 184	To study the financial burden of having head and neck cancer, and to explore its relation with health-related quality of life	The Social Difficulties Inventory (SDI) EORTC QLQ-C30, Cost of head and neck cancer questionnaire	The most notable costs of cancer that were a burden to patients were petrol (25%), home heating (24%), change in the type of food (21%), and loss of earnings (20%). During the previous week 15% had lost a moderate or large amount of income because of their medical condition. In terms of taking care of their financial needs, 10% were moderately dissatisfied and 15% very dissatisfied	8
Shankaran 2012 USA [46]	Cross-sectional	*N* = 284 Colon cancer (stage III)	19.7 months	To investigate factors associated with financial hardship in patients receiving adjuvant chemotherapy	Employment or income changes, insurance status and denials for specific services, copayments, deductibles, and monthly expenses, perceived financial burden	Thirty-eight percent of patients reported one or more financial hardships as a result of treatment. The factors most closely associated with treatment-related financial hardship were younger age and lower annual household income. Younger age, lower income, and unemployment or disability were most closely associated with treatment non-adherence	7
Sharp 2016 Ireland [41]	Cross-sectional	*N* = 740 Mixed	3–24 months	To identify factors associated with cancer-related financial stress and strain in breast and prostate cancer survivors in Ireland	Self-designed financial toxicity questionnaire including cancer-related OOP costs, objective and subjective financial difficulties.	Forty-eight percent reported cancer-related financial stress and 32% cancer-related financial strain and was more prominent among working individuals. Compared with those employed at diagnosis, risk of cancer-related financial stress was significantly lower in those not working (RR = 0.71, 95% CI 0.58–0.86) or retired (RR = 0.48, 95% CI 0.34–0.68).	8
Wheeler 2018 USA [35]	population-based prospective	*N* = 2.494 Breast cancer	5 and 25 months post diagnosis	To describe racial differences with regard to the financial impact of breast cancer	Self-designed financial toxicity questionnaire including job loss, income loss, financial and/or transportation barriers due to costs, refusal or delay in cancer treatment due to costs, loss of insurance	Since diagnosis, 58% of black women reported any adverse financial impact of cancer vs. 39% of white women (*p* < 0.001). In models adjusted for age, stage, and treatment, black women were more likely to report adverse financial impact due to cancer, including income loss, healthcare–related financial barriers, health care–related transportation barriers, and loss of health insurance	9
Authors/year/country	Study design	Sample size and population	Time since diagnosis	Objectives	Financial toxicity outcome measures used	Main findings	**Quality assessment**
Whitney 2016 USA [36]	Cross-sectional	*N* = 1.209 Mixed	Active treatment *N* = 166; <5 years after treatment *N* = 300; ≥ 5 years after treatment *N* = 487	To examine predictors of cancer-related financial difficulties and work modifications in a national sample of cancer survivors	Medical Expenditure Panel Survey Household Component and Experiences with Cancer Survivorship Supplement	Thirty-three percent reported any financial concern, 18% reporting financial difficulties like debt or bankruptcy. Forty-four made work modifications and 15% made long-term work modifications. Among survivors under age 65 years, predictors of long-term work modifications included good/fair/poor self-rated health, being married, uninsured, or publicly insured	7
Yabroff 2016 USA [43]	Cross-sectional population-based	*N* = 1.202 Mixed	Years since treatment; 1, *N* = 129 1- < 5, *N* = 137 ≥ 5, *N* = 214	To estimate the prevalence of financial hardship associated with cancer in the USA and identify characteristics associated with financial hardship	Medical Expenditure Panel Survey Experiences with Cancer questionnaire	Material financial hardship was more common in cancer survivors aged 18–64 years than in those ≥ 65 years (28.4% vs. 13.8%; *p* < 0.001). Survivors aged 18–64 years who were younger, female, nonwhite, and treated more recently and who had changed employment because of cancer were significantly more likely to report material financial hardship	8
Zheng 2016 USA [37]	Cross-sectional	*N* = 3.278 Mixed	Not reported	To provide nationally representative estimates of annual total economic burden among colorectal, female breast, and prostate cancer survivors by age group and cancer site	Medical Expenditure Panel Survey Household component	Compared with those without cancer, cancer survivors experienced annual excess medical expenditures. Nonelderly colorectal and breast cancer survivors experienced statistically significant annual excess employment disability (13.6% and 4.8%) and productivity loss at work (7.2 days and 3.3 days) and at home (4.5 days and 3.3 days). In contrast, elderly survivors had comparable productivity losses as those without cancer	7

*EORTC QLQ-C30*, European Organisation for Research and Treatment of Cancer Quality of Life Questionnaire Core 30; *HCT*, hematopoietic cell transplantation; *HRQOL*, health-related quality of life; *OOP*, out of pocket costs; *QoL*, Quality of life; *USA*, United States of America; *AUS*, Australia; *C.I.*, confidence interval; *FT*, financial toxicity; *OR*, odds ratio; *p*, probability; *RR*, relative risk

Both definitions and measures of financial toxicity varied strongly, and most measures were not validated making comparison between studies difficult. Some studies measured financial toxicity by the presence of consequences of increased costs and decreased income (e.g., bankruptcy, borrowing money, or debt) [24, 29, 38, 43]. Others measured financial toxicity by examining OOP costs [16, 21], decreased income [8, 19, 20, 25–27, 31, 32, 34, 36, 42, 46], the COST tool [40, 43, 45], the Goosens’ cost diary [19], the Breast Cancer Finances Survey [19], the EORTC QLQ-C30 [18, 28, 33, 34], the Financial Distress/Financial Well-Being scale [21], and by using questionnaires with self-designed questions. Only two studies objectively assessed financial toxicity [27, 42]. Employment status was measured as either unemployment/ceasing working or changes to employment such as a reduction in work hours.

### Quality of studies

The quality of 13 studies included in the review was arbitrarily rated as high, while 16 studies were rated as adequate quality and two as having a low quality (Table 2). The primary limitations of the studies were the lack of information about the degree of selection of the sample (e.g., whether there is a selective response), the cross-sectional research designs, and the lack of a validated financial toxicity measure and/or lack of objectively assessed financial toxicity.

### Financial toxicity and employment among cancer survivors versus a normative population

Four studies were identified that compared employment between those with a cancer diagnosis and those who have not had cancer [8, 20, 24, 37]. The results of 3 cross-sectional American studies showed that, among those < 64 years of age, being actively treated for cancer decreased the probability of employment [20], and increased employment disability [8], the number of missed workdays per year [8, 20, 37], the number of days spend in bed [8, 37], and the mean annual medical expenditures [8, 20, 37], compared with those not having cancer. A longitudinal Australian study compared financial strain between cancer survivors and the general population and concluded that although financial strain was higher in survivors compared with controls 6 months after diagnosis, it eased and was comparable with the general population at 12 months post-diagnosis [24].

### The relationship between financial toxicity and employment

The effect of cancer on financial toxicity and employment among cancer survivors was examined in all studies. The quantitative results are summarized in Table 2. Increased financial toxicity was associated with both unemployment, changed or reduced employment, lost days at work, or poor work ability in almost all included studies [8, 18–22, 24–30, 32–34, 38, 40, 42, 43, 45, 46]. However, a single study from Ireland identified employed individuals at greater risk for financial toxicity since they are more likely to experience a drop in income due to cancer [41]. Measures of financial toxicity varied strongly in these studies.

Examining only those studies that measured the impact of unemployment or ceasing work on financial toxicity identified twelve studies [18, 21, 24, 29, 30, 33, 38, 40, 45–47]. Half of the studies examining the impact of unemployment or ceasing work on financial toxicity have been conducted in the USA [24, 29, 30, 38, 45–47], only two conducted in Australia [23, 24], and one in Germany [18], the Netherlands [33], Iran [21], and Japan [40]. Across cancer types, those who were unemployed or ceased employment experienced greater financial toxicity [18, 21, 24, 29, 33, 40, 45, 46], objective financial burden (e.g., large decrease in income, selling/second mortgage on home, withdrawing money from retirement accounts, or bankruptcy) [29, 38, 48], or expenses [30] than patients who remained employed following their diagnosis. In contrast, an Irish study among breast and prostate cancer survivors reported that those who were not working had a significantly lower risk of cancer-related financial stress compared with those working (relative risk = 0.71, 95%CI 0.58–0.86) [41]. A study from the USA including a mixed group of cancer survivors concluded that survivors employed at diagnosis who took extended leave or switched to part-time work were more likely to report financial hardship (49%) compared with those employed that did not make changes (20%) and those who were not employed at diagnosis (17%) [43]. One study reported that unemployment was significantly associated with financial hardship while retirement was associated with decreased odds of financial hardship [46].

### Employment factors associated with financial toxicity

Studies analyzing employment factors associated with financial toxicity showed that those experiencing less financial toxicity had the following characteristics: paid leave [17], those who returned completely to work [21], not working [41], retired [41], privately insured [41], and those with higher household savings. Also, a higher age at diagnosis [40], being white [36, 43], a longer time since diagnosis [23, 38, 43], a lower disease stage [35], and a higher educational level [33, 39, 40] decreased the chance of financial toxicity.

In contrast, those unemployed [29, 33, 38], having to quit a job [18], taking a new job [18], retire [40], or with a reduction in work hours [18, 24, 44] because of cancer, those with non-regular employment [40], with part-time employment at diagnosis [44], and those with suboptimal workability [28] reported more financial toxicity. For those unemployed, a longer time since diagnosis was associated with a lower risk of financial toxicity but not among those who were employed [33]. Also, individuals reporting higher wage losses who had lower annual income [35, 36, 38, 41, 44–46], a low socioeconomic status [33], public insurance [35, 38, 41], poor insurance coverage [29], lack of substantial prescription drug coverage [44], experienced higher wage losses [42], or were uninsured [35, 38] reported more financial toxicity. Moreover, those who were younger [29, 31, 33, 35, 38, 39, 43, 44, 46, 49], being male [33, 41], or female [43], black [35, 39], Spanish-speaking Latinas [44], unmarried [33, 45], had dependents [41], residing in a non-metropolitan service area [36], with a mortgage/personal loan [41], with higher direct OOP costs [41], and increased household bills [41] reported more financial toxicity. Also those having two or more cancer diagnoses [38], a recurrence [44], noninvasive cancer [39], chemotherapy [22, 35, 44], lymphedema [19], lower physical [29, 49], mental [29] and socioemotional functioning limitations [49], and a lower quality of life [33] reported more financial toxicity. No studies analyzed confounders of the association between financial toxicity and employment.

## Discussion

This literature review identified a modest number of studies examining the relationship between financial toxicity and employment indicating relative scarcity of data on this subject. In general, cancer survivors can lose their job, they may have limitations in the amount or kind of work, they can experience job lock (not being able to take promotions or switch jobs) due to concerns of changing healthcare insurance, and they can experience higher cost-sharing when insured (especially in the USA) which can all contribute to financial toxicity. More research in this area is warranted since data varies between countries according to differences in healthcare and health insurance systems.

Unemployment, changed or reduced employment, lost days at work, or poor workability and changes to employment were associated with a higher risk of financial toxicity. However, a single study identified employment as a risk factor for financial toxicity among breast and prostate cancer survivors in Ireland [41]. This finding may reflect differences in health and social care systems [41]. In Ireland, the healthcare system consists of both private and public systems with an income limit determining acceptability for public services [50]. Those that are above the income limit are not accepted for public services and therefore pay for private healthcare. People with private care had higher costs compared with those in the public system, which suggests that employed individuals may be more susceptible to greater healthcare costs and therefore financial strain.

The relationship between negative work changes, and financial toxicity can be partly explained by the link between employment and health insurance. In some countries, like the USA, health insurance is often closely linked with employment. Therefore, losing one’s job because of cancer entails losing one’s health insurance. These two factors combined are a major risk factor for financial toxicity. However, some studies showed a negative association between work changes and financial toxicity in the setting of the universal healthcare coverage [24, 33]. This suggests that the association of employment and financial toxicity is not only a function of health care insurance but of social security systems as well. However, health insurance has an important role since those with private health insurance and paid leave often experienced a lower risk of financial toxicity while those with public insurance, those uninsured, those with poor insurance coverage, and those with a lack of substantial prescription drug coverage reported a higher risk of financial toxicity.

Differences between countries in employment and financial toxicity can also be caused by “return to work after cancer” policies. Return to work is influenced by social security systems, especially the length of paid sick leave. Furthermore, differences in legislation, incentives, and possibilities of an employer to provide employees with return to work programs differ among countries.

Besides negative work changes, being younger, non-white, unmarried, of lower education status, and residing with dependents or in non-metropolitan service areas were predictive of a higher risk of financial toxicity. Other factors associated with a higher risk of financial toxicity were having lower incomes, low socioeconomic status, a mortgage/personal loans, higher OOP costs and household bills, non-optimal health insurance, lower functioning and quality of life, and being more severely ill or on active treatment. This is not surprising since financial toxicity is a burden often affecting those most disadvantaged. These people often have fewer financial reserves or support on which to draw in times of unexpected financial strain. Also, these factors are often negatively associated with employment as well and therefore may have a compound effect on the likelihood of financial toxicity. Addressing financial toxicity may assist in addressing issues of access to care, equity of care, and may have significant impact on outcomes.

Only four studies compared survivors with a normative population. Three cross-sectional studies from the USA concluded that being actively treated for cancer had serious negative consequences regarding employment and medical expenditure. However, one longitudinal Australian study reported differences in financial strain at 6 months but no differences at 12 months after diagnosis. Time since diagnosis is thus an important is variable to consider but not all studies take this into consideration.

This systematic review has several strengths including a broad search of multiple keywords and search terms across various databases. The quality of most of the studies, as rated by a well-validated tool, was moderate to high. There were also a number of limitations to our study, which should be considered. We specifically targeted studies of adult cancer survivors excluding parents, siblings, caregivers, and spouses of cancer survivors. This has restricted the extent to which household financial toxicity can be examined and its relation to employment, although the impacts of financial toxicity are seen to extend to the parents, spouses, and caregivers of survivors [42]. Also, we did not include fully qualitative studies. In addition, we only focused on English language literature. Moreover, most studies were from a selected number of countries which limit generalizability across other countries or healthcare systems. Despite these limitations, this review is the first to explore the relationship between financial toxicity and employment among cancer survivors.

cThis review demonstrates the relative paucity of studies in the area of financial toxicity and employment and highlights a need for further research into the variables that are associated with the relationship between financial toxicity and employment to inform development of interventions to reduce financial toxicity because of employment change. For instance, the variation by cancer type, treatment type(s), duration of treatment(s), healthcare provider, and the role of community, state, and federal policy factors associated with financial hardship are still unclear. Further research should have a longitudinal design in order to focus on how the relationship between financial toxicity and employment changes over time. In addition, the use of a control group is warranted since financial problems can also occur due to other causes then cancer. In addition, the use of a validated financial toxicity measure and the use of a standard definition of financial toxicity will probably lead to results that can be more easily compared between studies.

In clinical practice, healthcare professionals should screen for financial toxicity during the disease trajectory. If financial toxicity is detected, directing patients to financial resources and advocating with an insurance company on behalf of the patient are possible actions one could take. Also, financial toxicity should be discussed with patients after diagnosis and regularly thereafter because it can influence treatment adherence and thus treatment efficacy. This is especially relevant in countries without universal healthcare coverage like the USA. In addition, healthcare professionals should have attention for the value of certain treatments in relation to their costs, and they should be prepared to discuss these tradeoffs with patients. This also implies that healthcare professionals’ should be informed on the OOP costs related to treatment. Finally, to decrease financial toxicity, patient should have basic knowledge on health insurance, potential costs of treatment, and available resources as well.

In conclusion, this review shows that financial toxicity is common after a cancer diagnosis but varies strongly between countries since it depends much upon the healthcare system. Researchers, healthcare professionals, health and safety officers in the work place, and patients themselves should all cooperate to tackle these complex issues.

## Abbreviations

MeSH: Medical Subject Heading
MM: Multiple myeloma
OOP: out-of-pocket
PRISMA: Preferred Reporting Items for Systematic Reviews and Meta-Analyses
QOL: Quality of life
(USA): United States of America

## References

[1] Zafar SY (2016). Financial toxicity of cancer care: It’s time to intervene. J Natl Cancer Inst.

[2] Gordon L, Merollini KM, Lowe A, Chan RJ (2016). A systematic review of financial toxicity among cancer survivors: we can’t pay the co-pay. Patient.

[3] Pisu M, Kenzik KM, Oster RA, Drentea P, Ashing KT, Fouad M, Martin MY (2015). Economic hardship of minority and non-minority cancer survivors 1 year after diagnosis: another long-term effect of cancer?. Cancer.

[4] Zafar SY, Peppercorn JM, Schrag D, Taylor DH, Goetzinger AM, Zhong X, Abernethy AP (2013). The financial toxicity of cancer treatment: a pilot study assessing out-of-pocket expenses and the insured cancer patient’s experience. Oncologist.

[5] McNulty J, Khera N (2015). Financial hardship—an unwanted consequence of cancer treatment. Curr Hematol Malign Rep.

[6] Ramsey SD, Bansal A, Fedorenko CR, Blough DK, Overstreet KA, Shankaran V, Newcomb P (2016). Financial insolvency as a risk factor for early mortality among patients with Cancer. J Clin Oncol.

[7] Knight TG, Deal AM, Dusetzina SB, Muss HB, Choi SK, Bensen JT, Williams GR (2018) Financial toxicity in adults with cancer: adverse outcomes and noncompliance. J Oncol Pract:JOP1800120. 10.1200/JOP.18.00120 10.1200/JOP.18.00120 30355027

[8] Guy GP, Ekwueme DU, Yabroff KR, Dowling EC, Li C, Rodriguez JL, de Moor JS, Virgo KS (2013). Economic burden of cancer survivorship among adults in the United States. J Clin Oncol.

[9] Altice CK, Banegas MP, Tucker-Seeley RD, Yabroff KR (2017). Financial hardships experienced by cancer survivors: a systematic review. J Natl Cancer Inst.

[10] Chan RJ, Gordon LG, Tan CJ, Chan A, Bradford NK, Yates P, Agbejule OA, Miaskowski C (2019). Relationships between financial toxicity and symptom burden in cancer survivors: a systematic review. J Pain Symptom Manag.

[11] Currow D, Aranda S (2016). Financial toxicity in clinical care today: a “menu without prices”1. Med J Aust.

[12] Tamminga SJ, de Boer AGEM, Verbeek JHAM, Frings-Dresen MHW (2010). Return-to-work interventions integrated into cancer care: a systematic review. Occup Environ Med.

[13] Mehnert A (2011). Employment and work-related issues in cancer survivors. Crit Rev Oncol Hematol.

[14] Liberati A, Altman DG, Tetzlaff J, Mulrow C, Gøtzsche PC, Ioannidis JPA, Clarke M, Devereaux PJ, Kleijnen J, Moher D (2009). The PRISMA statement for reporting systematic reviews and meta-analyses of studies that evaluate health care interventions: explanation and elaboration. PLoS Med.

[15] Mols F, Vingerhoets AJ, Coebergh JW, van de Poll-Franse LV (2005). Quality of life among long-term breast cancer survivors: a systematic review. Eur J Cancer.

[16] Beijers AJ, Mols F, Vreugdenhil G (2014). A systematic review on chronic oxaliplatin-induced peripheral neuropathy and the relation with oxaliplatin administration. Support Care Cancer.

[17] Albelda R, Wiemers E, Hahn T, Khera N, Salas Coronado DY, Abel GA (2019). Relationship between paid leave, financial burden, and patient-reported outcomes among employed patients who have undergone bone marrow transplantation. Qual Life Res.

[18] Arndt V, Koch-Gallenkamp L, Bertram H, Eberle A, Holleczek B, Pritzkuleit R, Waldeyer-Sauerland M, Waldmann A, Zeissig SR, Doege D, Thong MSY, Brenner H (2019). Return to work after cancer. A multi-regional population-based study from Germany. Acta Oncol.

[19] Dean LT, Moss SL, Ransome Y, Frasso-Jaramillo L, Zhang Y, Visvanathan K, Nicholas LH, Schmitz KH (2019). “it still affects our economic situation”: long-term economic burden of breast cancer and lymphedema. Support Care Cancer.

[20] Finkelstein EA, Tangka FK, Trogdon JG, Sabatino SA, Richardson LC (2009). The personal financial burden of cancer for the working-aged population. Am J Manag Care.

[21] Ghasempour M, Rahmani A, Davoodi A, Sheikhalipour Z, Ziaeei JE, Abri F (2015). Return to work and its relation to financial distress among Iranian cancer survivors. Asian Pac J Cancer Prev.

[22] Goodwin JA, Coleman EA, Sullivan E, Easley R, McNatt PK, Chowdhury N, Stewart CB (2013). Personal financial effects of multiple myeloma and its treatment. Cancer Nurs.

[23] Gordon LG, Walker SM, Mervin MC, Lowe A, Smith DP, Gardiner RA, Chambers SK (2017). Financial toxicity: a potential side effect of prostate cancer treatment among Australian men. Eur J Cancer Care (Engl).

[24] Gordon LG, Beesley VL, Mihala G, Koczwara B, Lynch BM (2017) Reduced employment and financial hardship among middle-aged individuals with colorectal cancer. Eur J Cancer Care (Engl) 26(5). 10.1111/ecc.12744 10.1111/ecc.12744 28771857

[25] Gordon L, Scuffham P, Hayes S, Newman B (2007). Exploring the economic impact of breast cancers during the 18 months following diagnosis. Psychooncology.

[26] Greaves-Otte JG, Greaves J, Kruyt PM, van Leeuwen O, van der Wouden JC, van der Does E (1991). Problems at social re-integration of long-term cancer survivors. Eur J Cancer.

[27] Guerin A, Sasane M, Dea K, Zhang J, Culver K, Nitulescu R, Wu EQ, Macalalad AR (2016). The economic burden of brain metastasis among lung cancer patients in the United States. J Med Econ.

[28] Ho PJ, Hartman M, Gernaat SAM, Cook AR, Lee SC, Hupkens L, Verkooijen HM (2018). Associations between workability and patient-reported physical, psychological and social outcomes in breast cancer survivors: a cross-sectional study. Support Care Cancer.

[29] Khera N, Chang YH, Hashmi S, Slack J, Beebe T, Roy V, Noel P, Fauble V, Sproat L, Tilburt J, Leis JF, Mikhael J (2014). Financial burden in recipients of allogeneic hematopoietic cell transplantation. Biol Blood Marrow Transplant.

[30] Massa ST, Osazuwa-Peters N, Adjei Boakye E, Walker RJ, Ward GM (2019). Comparison of the financial burden of survivors of head and neck cancer with other cancer survivors. JAMA Otolaryngol Head Neck Surg.

[31] Nekhlyudov L, Walker R, Ziebell R, Rabin B, Nutt S, Chubak J (2016). Cancer survivors’ experiences with insurance, finances, and employment: results from a multisite study. J Cancer Surviv.

[32] Paul C, Boyes A, Hall A, Bisquera A, Miller A, O'Brien L (2016). The impact of cancer diagnosis and treatment on employment, income, treatment decisions and financial assistance and their relationship to socioeconomic and disease factors. Support Care Cancer.

[33] Pearce A, Tomalin B, Kaambwa B, Horevoorts N, Duijts S, Mols F, van de Poll-Franse L, Koczwara B (2019). Financial toxicity is more than costs of care: the relationship between employment and financial toxicity in long-term cancer survivors. J Cancer Surviv.

[34] Rogers SN, Harvey-Woodworth CN, Hare J, Leong P, Lowe D (2012). Patients' perception of the financial impact of head and neck cancer and the relationship to health related quality of life. Br J Oral Maxillofac Surg.

[35] Wheeler SB, Spencer JC, Pinheiro LC, Carey LA, Olshan AF, Reeder-Hayes KE (2018). Financial impact of breast cancer in black versus white women. J Clin Oncol.

[36] Whitney RL, Bell JF, Reed SC, Lash R, Bold RJ, Kim KK, Davis A, Copenhaver D, Joseph JG (2016). Predictors of financial difficulties and work modifications among cancer survivors in the United States. J Cancer Surviv.

[37] Zheng Z, Yabroff KR, Guy GP, Han X, Li C, Banegas MP, Ekwueme DU, Jemal A (2016). Annual medical expenditure and productivity loss among colorectal, female breast, and prostate cancer survivors in the United States. J Natl Cancer Inst.

[38] Banegas MP, Guy GP, de Moor JS, Ekwueme DU, Virgo KS, Kent EE, Nutt S, Zheng Z, Rechis R, Yabroff KR (2016). For working-age cancer survivors, medical debt and bankruptcy create financial hardships. Health Aff (Millwood).

[39] Casilla-Lennon MM, Choi SK, Deal AM, Bensen JT, Narang G, Filippou P, McCormick B, Pruthi R, Wallen E, Tan HJ, Woods M, Nielsen M, Smith A (2018). Financial toxicity among patients with bladder cancer: reasons for delay in care and effect on quality of life. J Urol.

[40] Honda K, Gyawali B, Ando M, Kumanishi R, Kato K, Sugiyama K, Mitani S, Masuishi T, Narita Y, Bando H, Taniguchi H, Kadowaki S, Ura T, Muro K (2019). Prospective survey of financial toxicity measured by the comprehensive score for financial toxicity in Japanese patients with cancer. J Glob Oncol.

[41] Sharp L, Timmons A (2016). Pre-diagnosis employment status and financial circumstances predict cancer-related financial stress and strain among breast and prostate cancer survivors. Support Care Cancer.

[42] Lauzier S, Levesque P, Mondor M, Drolet M, Coyle D, Brisson J, Masse B, Provencher L, Robidoux A, Maunsell E (2013). Out-of-pocket costs in the year after early breast cancer among Canadian women and spouses. J Natl Cancer Inst.

[43] Yabroff KR, Dowling EC, Guy GP, Banegas MP, Davidoff A, Han X, Virgo KS, McNeel TS, Chawla N, Blanch-Hartigan D, Kent EE, Li C, Rodriguez JL, de Moor JS, Zheng Z, Jemal A, Ekwueme DU (2016). Financial hardship associated with cancer in the United States: findings from a population-based sample of adult cancer survivors. J Clin Oncol.

[44] Jagsi R, Pottow JA, Griffith KA, Bradley C, Hamilton AS, Graff J, Katz SJ, Hawley ST (2014). Long-term financial burden of breast cancer: experiences of a diverse cohort of survivors identified through population-based registries. J Clin Oncol.

[45] Huntington SF, Weiss BM, Vogl DT, Cohen AD, Garfall AL, Mangan PA, Doshi JA, Stadtmauer EA (2015). Financial toxicity in insured patients with multiple myeloma: a cross-sectional pilot study. Lancet Haematol.

[46] Shankaran V, Jolly S, Blough D, Ramsey SD (2012). Risk factors for financial hardship in patients receiving adjuvant chemotherapy for colon cancer: a population-based exploratory analysis. J Clin Oncol.

[47] Guy GP, Yabroff KR, Ekwueme DU, Rim SH, Li R, Richardson LC (2017). Economic burden of chronic conditions among survivors of cancer in the United States. J Clin Oncol.

[48] Guy GP, Yabroff KR, Ekwueme DU, Virgo KS, Han X, Banegas MP, Soni A, Zheng Z, Chawla N, Geiger AM (2015). Healthcare expenditure burden among non-elderly cancer survivors, 2008-2012. Am J Prev Med.

[49] Rogers SN, Harvey-Woodworth CN, Lowe D (2012). Patients’ perspective of financial benefits following head and neck cancer in Merseyside and Cheshire. Br J Oral Maxillofac Surg.

[50] van Doorslaer E, Wagstaff A (1992). Equity in the delivery of health care: some international comparisons. J Health Econ.

